# Evaluation of polymyxin B AUC/MIC ratio for dose optimization in patients with carbapenem-resistant *Klebsiella pneumoniae* infection

**DOI:** 10.3389/fmicb.2023.1226981

**Published:** 2023-08-22

**Authors:** Peile Wang, Shaohua Liu, Guangzhao Qi, Min Xu, Tongwen Sun, Jing Yang

**Affiliations:** ^1^Department of Pharmacy, The First Affiliated Hospital of Zhengzhou University, Zhengzhou, China; ^2^Henan Key Laboratory of Precision Clinical Pharmacy, Zhengzhou University, Zhengzhou, China; ^3^Henan Engineering Research Center for Application and Translation of Precision Clinical Pharmacy, Zhengzhou University, Zhengzhou, China; ^4^Department of General Intensive Care Unit, The First Affiliated Hospital of Zhengzhou University, Zhengzhou, China; ^5^Department of Clinical Laboratory, The First Affiliated Hospital of Zhengzhou University, Zhengzhou, China

**Keywords:** Carbapenem-resistant *Klebsiella pneumonia*, polymyxin B, antimicrobial susceptibility testing, AUC/MIC, Monte Carlo simulation

## Abstract

Polymyxin B has been used as a last-line therapy for the treatment of carbapenem-resistant gram-negative bacterial infection. The pharmacokinetic/pharmacodynamic index (AUC/MIC) of polymyxin B has not been clinically evaluated, given that the broth microdilution method for polymyxin susceptibility testing is rarely used in hospitals. This study analyzed data from 77 patients with carbapenem-resistant *Klebsiella pneumoniae* infections. Among the samples, 63 *K. pneumoniae* isolates had MIC values of 1.0 mg/L as measured by broth microdilution but 0.5 mg/L as measured using the Vitek 2 system. Polymyxin B AUC/MIC was significantly associated with clinical response (*p* = 0.002) but not with 30-day all-cause mortality (*p* = 0.054). With a target AUC/MIC value of 50, Monte Carlo simulations showed that a fixed dose of 100 mg/12 h and three weight-based regimens (1.25 mg/kg/12 h for 80 kg and 1.5 mg/kg/12 h for 70 kg/80 kg) achieved a cumulative fraction of response >90% regardless of renal function, but the risk of nephrotoxicity was high. For patients with carbapenem-resistant *K. pneumoniae* infections, the underestimation of polymyxin resistance in automated systems need to be taken into account when optimizing polymyxin B dosing based on pharmacokinetic/pharmacodynamic principles.

## 1. Introduction

Infections caused by antimicrobial resistance isolates are a major threat to public health (Algammal et al., 2023). Carbapenem-resistant gram-negative bacteria (CR-GNB) are the main contributors to infectious diseases caused by multidrug-resistant bacteria (Karampatakis et al., 2023). Polymyxins (i.e., colistin and polymyxin B) have been used as alternatives for the treatment of infections caused by CR-GNB. Unfortunately, these drugs have wide inter-individual variability in their pharmacokinetics (PK) and a narrow therapeutic index (Nang et al., 2021).

Recent studies have revealed that the ratio of the free-drug area under the concentration–time curve to the minimum inhibitory concentration (fAUC/MIC) is the most predictive PK/pharmacodynamics (PK/PD) index for polymyxin B (Tsuji et al., 2019). In our previous study, we demonstrated that an AUC_ss,24h_ threshold of 50–100 mg·h/L was a good predictor of clinical response and acute kidney injury in a real-world cohort of patients treated with polymyxin B for CR-GNB infections. However, MIC values were not available because an automated system was used (Yang et al., 2022).

Broth microdilution (BMD) assay is a reference method recommended by the Clinical and Laboratory Standards Institute (CLSI) and the European Committee on Antimicrobial Susceptibility Testing (EUCAST) for antimicrobial susceptibility testing (AST) of polymyxins (Satlin et al., 2020), but automated systems are more commonly used in clinical laboratories. At our hospital, the MICs of all included isolates were ≤0.5 mg/L (Yang et al., 2022). Considering that automated systems may underestimate MIC for polymyxins (Pfennigwerth et al., 2019; Zhu et al., 2021), this study aimed to measure the MIC of polymyxins via BMD, in order to evaluate the correlation between AUC/MIC ratio in polymyxin B and clinical outcome in patients with Carbapenem-resistant *Klebsiella pneumoniae* (CRKP) infections, and to explore optimal dosing regimens using Monte Carlo simulations based on PK/PD target.

## 2. Materials and methods

### 2.1. Study design

Data were derived from a previous retrospective study conducted at the First Affiliated Hospital of Zhengzhou University from April 2018 to March 2022 (Yang et al., 2022). This study was approved by the Ethics Committees of the First Affiliated Hospital of Zhengzhou University (No. 2020-KY-0318) and was registered with the Chinese Clinical Trial Register (No. ChiCTR2100043208).

Patients with CRPK infection whose strains were collected before polymyxin B treatment were included in this study. The primary endpoints were clinical response and 30-day all-cause mortality (Yang et al., 2022). Clinical response was considered at the end of treatment by two physicians: disappearance or improvement of clinical symptoms (body temperature < 38.0°C), radiological resolution of signs of infection, and improved biochemical indicators of infection (≥30% decrease in total peripheral white blood cell count or C-reactive protein level). Patients who did not meet all the above criteria were classified as cases of clinical failure.

### 2.2. Microbiology

A total of 63 bronchoalveolar lavage fluid samples, 6 blood samples, 4 hydrothorax and as cite samples, 2 cerebrospinal fluid samples, and 2 skin tissue pus samples were collected. Species identification and AST were performed using a Vitek^®^ MS MALDI-TOF system (bioMérieux, Marcy-l’Etoile, France) and a Vitek^®^ 2 COMPACT automated system with Vitek^®^ 2 AST cards (0.5–16 mg/L of colistin), respectively. Polymyxin B reference MICs were performed retrospectively from frozen isolates on BMD panels (Wenzhou Kangtai Biotechnology Co., LTD, China). Briefly, 2-fold dilutions ranging from 0.25 to 32 mg/L of polymyxin B were prepared in 96-well plates, using a final inoculum of 5 × 10^5^ cfu/mL of each isolate in sterile water. *Escherichia coli* ATCC 25922 and *P. aeruginosa* ATCC 27853 were used as susceptible controls. CRKP was defined as cases in which *K. pneumoniae* was non-susceptible to at least one carbapenem antibiotic (CLSI, 2018). Carbapenem resistance was defined as an MIC breakpoint of ≥4 mg/L for meropenem/imipenem/doripenem or ≥2 mg/L for ertapenem (CLSI, 2020). Polymyxin breakpoints of susceptibility ≤2 mg/L/resistance >2 mg/L were applied based on United States Committee on Antimicrobial Susceptibility Testing (USCAST) criteria (CLSI, 2015).

Rates of essential agreement (EA), category agreement (CA), very major error (VME), and major error (ME) were estimated using BMD as the reference method. According to CLSI recommendations, a method must exhibit CA ≥ 90%, EA ≥ 90%, VME < 3%, and ME <3% in order to be considered acceptable (Pogue et al., 2020).

### 2.3. Pharmacokinetic analysis

As for the PK study, two blood samples were collected before infusion (C_0h_) and 2 h after the start of infusion (C_2h_), at least 3 days after polymyxin B treatment. Plasma concentrations were determined using a validated ultra-performance liquid chromatography–tandem mass spectrometry method previously published by our laboratory (Wang et al., 2020a).

AUC_ss,24h_ was estimated using the Bayesian priors of our previously published population PK model using the Phoenix^®^ NLME software package (v8.3, Pharsight, Mountain View, CA, United States) (Wang et al., 2020b). Monte Carlo simulations with 1,000 subjects were performed on fixed and weight-based regimens based on the population PK model. Loading dose was twice the maintenance dose, and infusion time was 1 h. The trapezoidal rule was used to calculate the AUC across 24 h.

### 2.4. Dosing simulations

Using an AUC/MIC of 50 as the PK/PD target, a probability of target attainment (PTA) value >90% was considered to represent effectiveness. The cumulative fraction of response (CFR) was the sum of the isolates’ frequency for each MIC multiplied by the PTA, and a value of 90% was considered to represent effectiveness. In addition, the probability (%) of achieving the target AUC (50–100 mg·h/L) was calculated, and an AUC of >100 mg·h/L was taken as a predictor of nephrotoxicity, according to international consensus guidelines (Tsuji et al., 2019).

### 2.5. Statistical analysis

Statistical analyses were performed using the Statistical Package for the Social Sciences version 26.0 (SPSS Inc., Chicago, IL, United States). Continuous variables are presented in the form of median (interquartile range, IQR) and were analyzed using the Mann–Whitney U test. Categorical variables are presented in the form of percentage/frequency (%) and were analyzed using the Chi-square test or Fisher’s exact test. Receiver operating characteristic (ROC) curves was used to explore the relationship between PK/PD index and outcome. A *p* value < 0.05 was considered statistically significant.

## 3. Results

### 3.1. Patient characteristics and susceptibility

A total of 77 patients were included for analysis (Table 1). All isolates were susceptible to polymyxin B. The MIC by BMD was 1.0 mg/L for 63 isolates, 0.5 mg/L for 10, 2.0 mg/L for three, and 0.25 mg/L for one. The automated AST system showed acceptable levels of CA (100%), EA (96.1%), ME (0%), and VME (0%). In addition, results on the susceptibility of CRKP isolates to antimicrobials are shown in Supplementary Table S1.

**Table 1:** Patient characteristics.

Parameter	Value (*n* = 77)
Age, years	57.0 (48.5–67.0)
Gender	
Male, *n* (%)	57 (74.0%)
Female, *n* (%)	20 (26.0%)
Weight, kg	70.0 (60.0–75.0)
BMI, kg/m^2^	23.1 (20.00–24.9)
ICU admission, *n* (%)	76 (98.7)
Mechanical ventilation, *n* (%)	54 (70.1%)
Septic shock, *n* (%)	30 (39.0%)
APACHE score	20.0 (17.0–23.0)
SOFA score	7.0 (6.0–10.0)
Comorbidities, *n* (%)	
Diabetes	20 (26.0%)
Hypertension	38 (49.4%)
Malignancy	9 (11.7%)
Chronic kidney disease	6 (7.8%)
Laboratory data	
Albumin, g/L	30.7 (28.1–33.8)
Serum creatinine, μmol/L	59.0 (42.3–77.5)
GFR, mL/min·1.73 m^2^	104.7 (78.3–121.6)
White blood cells, 10^9^/L	11.2 (8.4–14.0)
Platelets, 10^9^/L	179.0 (108.5–285.0)
C-reactive protein, μg/L	61.2 (31.8–158.1)
Procalcitonin, ng/mL	0.9 (0.3–3.0)
Polymyxin B treatment	
Duration, days	11.0 (6.0–14.0)
Daily dose, mg	150.0 (100.0–150.0)
Daily dose/weight, mg/kg/day	1.9 (1.5–2.3)
AUC_ss,24 h_, mg·h/L	54.3 (36.3–71.6)
Concomitant antibiotics, *n* (%)	
Carbapenem	21
Cephalosporin	18
Tigecycline	24
Carbapenem + Tigecycline	10
Cephalosporin + Tigecycline	4

Data are reported in the form *n* (%) or median (interquartile range, IQR). BMI, body mass index; APACHE II score, Acute Physiology and Chronic Health Evaluation II; SOFA, Sequential Organ Failure Assessment; GFR, glomerular filtration rate.

### 3.2. Clinical outcomes with AUC/MIC

**Figure 1:**
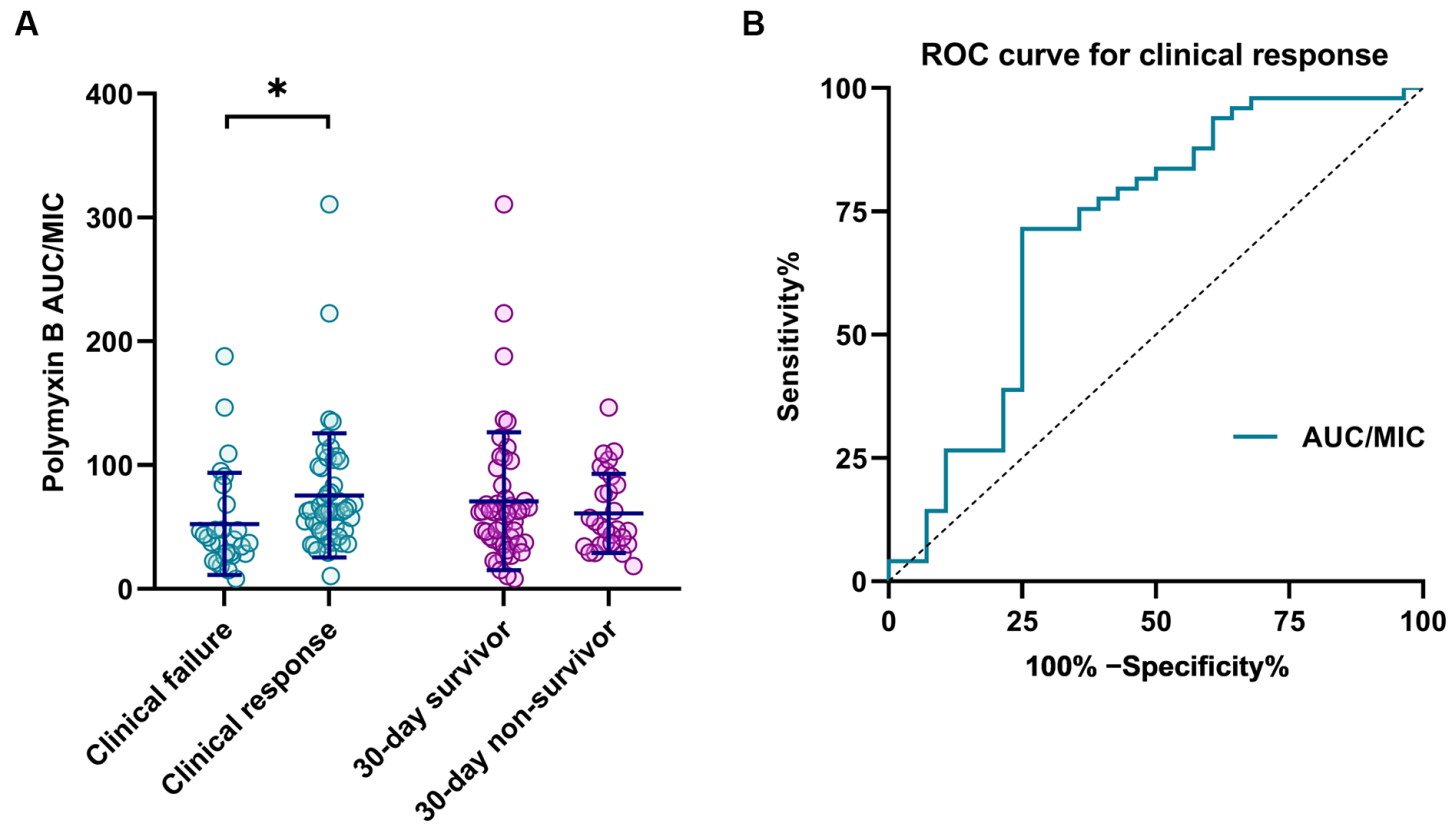
**(A)** Correlation of polymyxin B clinical outcome with PK/PD parameters (AUC/MIC). *p < 0.05. **(B)** ROC curve for polymyxin B PK/PD as a predictor of clinical response.

At the end of polymyxin B treatment, a clinical response was observed in 49 cases (63.6%). Patients with clinical failure had a lower median AUC/MIC than those who exhibited a clinical response (*p* = 0.002; Figure 1A). Among all patients, 30-day all-cause mortality was 37.7% (29/77), with and the median survival time among patients who did not survive was 9 days (IQR 5-17.5). No significant difference in AUC/MIC between survivors and non-survivors was observed (*p* = 0.054; Figure 1A).

The area under the ROC curve for AUC/MIC as a predictor of clinical response was 0.714 (95% CI 0.583–0.844; *p* = 0.002; Figure 1B). The optimal cut-off, at the maximum Youden index (0.464), corresponded to an AUC/MIC value of 49.3, with predictive sensitivity and specificity of 71.4 and 73.5%, respectively.

In addition, several potential risk factors that may have affected clinical outcomes were evaluated (Table 2). Platelets, occurrence of septic shock, mechanical ventilation, and AUC were independently associated with clinical response and mortality (*p* < 0.05). AUC/MIC and AUC/MIC ≥50 only had significant association with clinical response (*p* < 0.05). Furthermore, sample type had no significant association with clinical outcome (Supplementary Table S2).

**Table 2:** Univariate analysis for clinical response and 30-day all-cause mortality.

Variable	Response (*n* = 49)	Failure (*n* = 28)	*p*	Survival (*n* = 48)	No survival (*n* = 29)	*p*
Age, years	59.0 (50.0–66.0)	57.5 (52.5–70.0)	0.327	60.0 (55.0–65.0)	55.5 (45.5–68.0)	0.858
Gender, *n* male	37 (75.5%)	20 (71.4%)	0.694	39 (81.3%)	18 (62.1%)	0.063
Weight, kg	70.0 (60.0–73.0)	70.0 (64.5–75.0)	0.339	70.0 (60.0–75.0)	65.3 (60.0–72.5)	0.480
BMI, kg/m^2^	22.9 (19.8–24.8)	23.7 (22.0–25.0)	0.454	23.1 (20.8–24.9)	23.3 (18.9–24.2)	0.462
SOFA score	7.0 (6.0–10.0)	8.0 (5.5–9.5)	0.342	7.0 (5.0–9.0)	9.0 (7.0–10.5)	0.060
APACHE II score	20.0 (17.0–22.0)	20.0 (17.0–26.0)	0.274	20.0 (18.0–22.0)	20.0 (17.0–24.0)	0.817
Septic shock	14 (28.6%)	16 (57.1%)	0.013	9 (18.8%)	21 (72.4%)	<0.001
Mechanical ventilation	29 (59.2%)	25 (89.3%)	0.005	29 (60.4%)	25 (86.2%)	0.017
**Laboratory data**						
GFR, mL/min⋅1.73m^2^	105 (92.0–119)	105 (55.4–121)	0.380	106 (97.8–120)	90.49 (59.3–116)	0.050
Albumin, g/L	30.7 (27.5–33.0)	29.2 (28.1–31.2)	0.751	30.1 (28.1–33.0)	30.6 (28.0–32.1)	0.458
Platelets, 10^9^/L	208 (151–322)	143 (79.0–191)	0.030	242 (172–322)	143 (78.0–168)	<0.001
CRP, μg/L	58.7 (26.1–109)	118 (44.1–175)	0.151	60.3 (27.2–150)	94.1 (44.1–159)	0.245
Procalcitonin, ng/mL	0.41 (0.23–1.68)	1.29 (0.36–3.96)	0.138	0.41 (0.23–1.76)	1.02 (0.39–4.79)	0.293
**Polymyxin B treatment**						
Dose/weight, mg/kg/d	2.14 (1.77–2.50)	1.58 (1.43–2.01)	0.001	2.06 (1.43–2.50)	1.82 (1.55–2.27)	0.371
AUC_ss,24h_	62.8 (47.0–77.7)	36.6 (27.6–60.5)	0.001	69.9 (40.5–92.6)	47.9 (31.2–65.8)	0.024
AUC/MIC	63.3 (47.0–103)	38.6 (28.8–76.0)	0.002	62.9 (35.4–101)	49.3 (36.1–87.5)	0.054
AUC/MIC ≥50	35 (71.4%)	7 (25.0%)	< 0.001	27 (56.3%)	15 (51.7%)	0.772

GFR, glomerular filtration rate; CRP, C-reactive protein; AUC_ss,24h_, area under the curve over 24 h at steady state; MIC, minimum inhibitory concentration.

### 3.3. Dosing simulations

**Figure 2:**
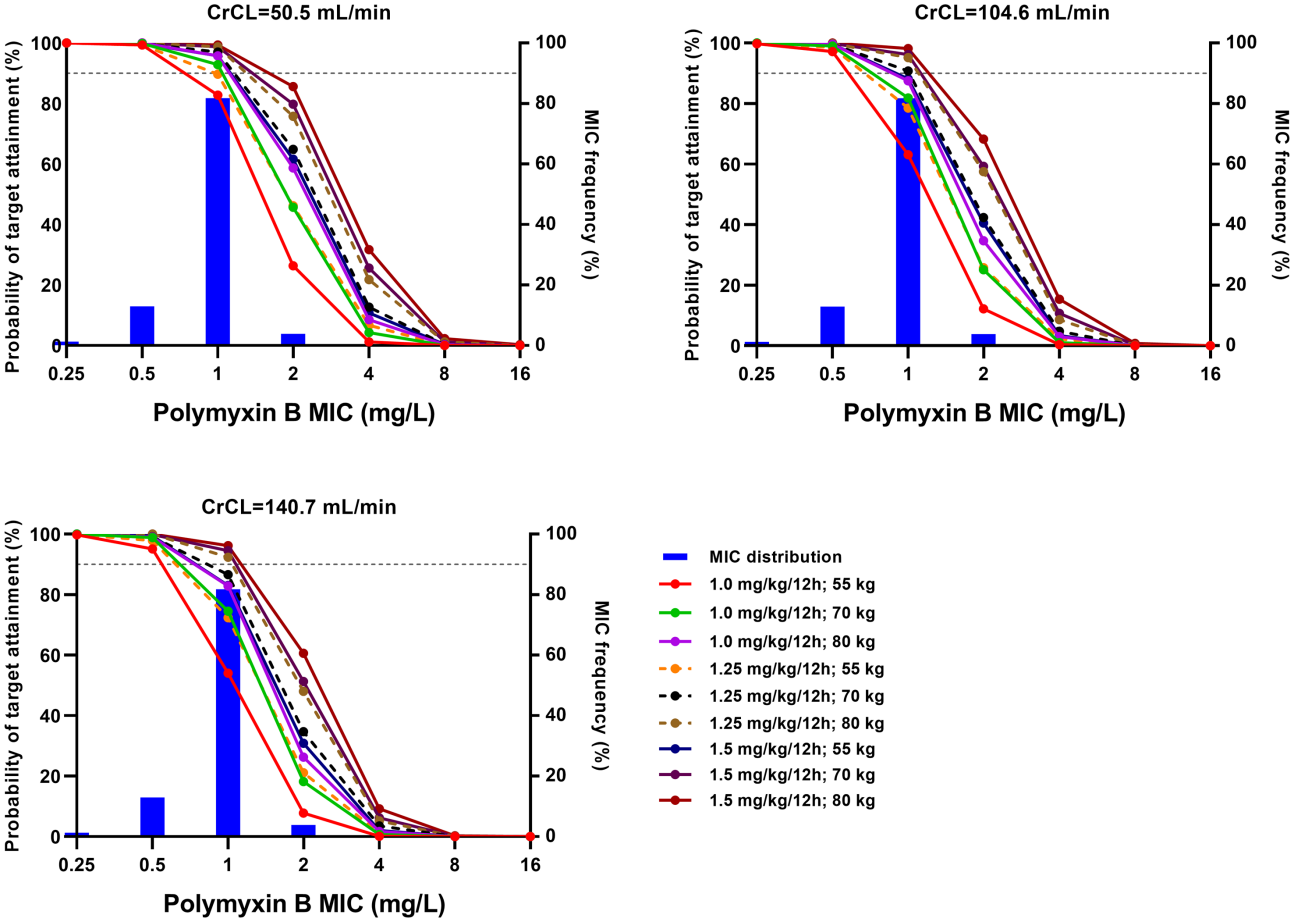
Probability of target attainment (PTA) for various regimens with different creatinine clearance (CrCL). The target was an area under the curve/minimum inhibitory concentration (AUC/MIC) ≥ 50. Histograms represent the frequency distribution as established by broth microdilution. Horizontal dotted lines represent 90% PTA.

Taking AUC/MIC ≥ 50 as the PK/PD target, PTAs for various regimens against MIC distribution are shown in Figure 2 and Supplementary Figure S1. Additionally, CFR, the probability of target AUC (50–100 mg·h/L), and nephrotoxicity (AUC > 100 mg·h/L) are shown in Table 3 and Supplementary Table S3.

**Table 3:** Probability of achievement of target AUC, PTA, and CFR for different polymyxin B regimens according to the 10th, 50th, and 90th percentiles of creatinine clearance (CrCL) and weight (Wt.).

CrCL (mL/min)	Dosing (mg/kg; q12h)	Wt. (kg)	Probability (%) of target AUC	Probability (%) of AUC > 100 mg·h/L	PTA for different MICs (mg/L)							CFR (%) for PK/PD target
					0.25	0.5	1	2	4	8	16	
50.5	1.0	55	56.4	26.4	100	99.4	82.8	26.4	1.2	0	0	83.0
		70	47.2	45.7	100	99.9	92.9	45.7	4.3	0	0	92.1
		80	37.1	58.7	100	100	95.8	58.7	8.5	0.3	0	95.0
	1.25	55	43.5	46.2	100	99.4	89.7	46.2	6.7	0	0	89.4
		70	32.1	64.9	100	99.9	97.0	64.9	12.7	0.4	0	96.2
		80	23.0	75.8	100	100	98.8	75.8	21.8	1.2	0	98.1
	1.5	55	34.2	61.6	100	99.9	95.8	61.6	10.8	0.4	0	95.1
		70	19.0	79.8	100	100	98.8	79.8	25.6	1.2	0	98.2
		80	13.8	85.6	100	100	99.4	85.6	31.7	2.3	0.3	98.9
104.6	1.0	55	50.9	12.2	99.8	97.2	63.1	12.2	0.4	0	0	66.0
		70	56.8	25.1	100	99.0	81.9	25.1	1.0	0	0	52.1
		80	52.8	34.7	100	99.6	87.5	34.7	3.0	0	0	87.2
	1.25	55	52.7	25.8	100	98.5	78.5	25.8	2.2	0	0	79.3
		70	48.4	42.4	100	99.6	90.8	42.4	4.8	0	0	90.2
		80	37.7	57.5	100	99.9	95.2	57.5	8.6	0.2	0	94.4
	1.5	55	47.7	40.5	100	99.6	88.2	40.5	3.4	0	0	88.0
		70	36.9	59.3	100	99.9	96.2	59.3	10.7	0.2	0	95.3
		80	29.9	68.3	100	100	98.2	68.3	15.4	0.8	0	97.3
140.7	1.0	55	46.2	7.8	99.8	95.1	54.0	7.8	0.1	0	0	58.1
		70	56.3	18.2	100	98.9	74.5	18.2	0.7	0	0	75.8
		80	56.6	26.3	100	99.0	82.9	26.3	1.7	0	0	83.0
	1.25	55	51.3	21.1	99.9	97.9	72.4	21.1	1.2	0	0	74.1
		70	51.9	34.7	100	99.1	86.6	34.7	3.5	0	0	86.4
		80	44.4	48.0	100	100	92.4	48.0	5.2	0.1	0	91.8
	1.5	55	52.1	30.9	100	99.4	83.0	30.9	2.0	0	0	83.3
		70	43.2	51.3	100	99.9	94.5	51.3	6.2	0.1	0	93.6
		80	35.6	60.6	100	100	96.2	60.6	9.2	0.3	0	95.4

Target AUC_24h_, 50–100 mg·h/L; PTA, probability of target attainment; CFR, cumulative fraction of response; PK/PD target, PK/PD ≥ 50.

## 4. Discussion

As manual preparation of BMD plates is extremely labor intensive, these have been almost entirely replaced by gradient strips or automated systems in clinical laboratories (Pfennigwerth et al., 2019). In this study, most (81.8%) polymyxin MICs of CRKP isolates tested using the Vitek 2 system were one-fold lower dilutions than BMD, which is consistent with other reports (Pfennigwerth et al., 2019; Zhu et al., 2021). Polymyxin B (CID: 9833652) and colistin (CID: 5311054) are lipopeptide components with large-molecule and amphiphilic properties [National Center for Biotechnology Information (NCBI), 2023]. These physicochemical characteristics can affect the accuracy of polymyxin MIC values measured via disk diffusion or using an automated system (Nang et al., 2021). Therefore, it is necessary to note that automated systems may underestimate MIC values when optimizing polymyxin B dose based on the PK/PD principle.

The current study showed that an AUC/MIC rate of >49.3 was significantly associated with clinical response, which was in line with the PK/PD index derived from murine infection models (Lakota et al., 2018) and the target AUC reported in our previous study (Yang et al., 2022). This may be because polymyxin MICs were mostly (81.8%) 1.0 mg/L. Nevertheless, the univariate analysis showed that AUC/MIC ≥50 had no correlation with mortality (*p* > 0.05). This can be attributed to the fact that patients with CRKP infections suffered from serious underlying diseases and were in poor physical condition, both of which are likely to have affected their clinical outcomes (Liu et al., 2023).

With a target AUC/MIC value of 50, Monte Carlo simulations showed that high dosage with low creatinine clearance (CrCL) resulted in a high PTA (Figure 2; Supplementary Figure S1). All regimens achieved >90% PTA at MICs ≤0.5 mg/L, and no regimen achieved >90% PTA at MICs ≥2.0 mg/L, which was in agreement with previous reports (Miglis et al., 2018; Xie et al., 2020; Wang et al., 2022; Yu et al., 2022). For MICs of 1.0 mg/L, a fixed regimen (100 mg/12 h) and three weight-based regimens (1.25 mg/kg for 80 kg/12 h, 1.5 mg/kg for 70 kg/12 h and 80 kg/12 h) achieved >90% PTA.

Given that accurate MIC values for polymyxins are not always available in clinical practice, CFR may be more useful than PTA for empirical dosing. As shown in Table 3 and Supplementary Table S3, only the above four regimens achieved a CFR > 90% against CRKP, regardless of renal function. However, these regimens also led to a high probability of AUC > 100 mg·h/L (at least 44.7%). Based on a population PK model of healthy Chinese subjects, Bian et al. suggested that polymyxin B dosing regimens of 1.0–1.5 mg/kg/12 h are appropriate for *K. pneumoniae* (Bian et al., 2021); however, this study did not consider toxic exposure and the differences between healthy subjects and patients in terms of PK characteristics.

The present study has several limitations. First, given that this was a single-center retrospective study with a small sample size, the power of the estimated PK/PD index in predicting clinical outcomes was limited, and risk factors for poor clinical outcome (such as underlying diseases, severity of illness, use of combination therapy, and variability in PK parameters and MICs) were not investigated. Second, only total-drug AUC was estimated in this study, and therefore the impact of free drug concentrations on PK/PD metrics needs to be further investigated. Third, *K. pneumoniae* isolates present resistance to antimicrobial agents via one or more mechanisms, including production of specified enzymes, decreased cell permeability through loss of OMPs, overexpression of efflux pumps, and modification of the target of the antimicrobial agent (Ahmadi et al., 2021, 2022; Karampatakis et al., 2023). Resistance gene testing can reveal the resistance characteristics and transmission trends of CRKP strains in the relevant region and assist in antibiotic treatment; this approach requires further in-depth research.

## 5. Conclusion

In conclusion, comparison of BMD and the Vitek 2 system indicated that the polymyxin MICs of CRKP might be underestimated by a one-fold level of dilution by the Vitek 2 system in our hospital. With a target AUC/MIC value of 50, empirical dosages of 100 mg/12 h, 1.25 mg/kg/12 h at 80 kg, or 1.5 mg/kg/12 h at both 70 kg and 80 kg could achieve effective therapeutic outcomes, but efficacy needs to be balanced against the potential for nephrotoxicity.

## Data availability statement

The original contributions presented in the study are included in the article/Supplementary material, further inquiries can be directed to the corresponding authors.

## Ethics statement

The studies involving human participants were reviewed and approved by the Ethics Committees of the First Affiliated Hospital of Zhengzhou University. The ethics committee waived the requirement of written informed consent for participation.

## Author contributions

PW and SL contributed to the data acquisition, analysis, and interpretation. PW contributed to manuscript preparation. GQ performed the experiments. JY supervised the research and revised the manuscript. MX and TS designed the study. All authors contributed to the article and approved the submitted version.

## Funding

This work was supported by the National Key R&D Program of China (Grant No. 2020YFC2008304) and the Educational Committee of Henan Province (23A350006).

## Conflict of interest

The authors declare that the research was conducted in the absence of any commercial or financial relationships that could be construed as a potential conflict of interest.

## Publisher’s note

All claims expressed in this article are solely those of the authors and do not necessarily represent those of their affiliated organizations, or those of the publisher, the editors and the reviewers. Any product that may be evaluated in this article, or claim that may be made by its manufacturer, is not guaranteed or endorsed by the publisher.
