# Supplementary material for: Evaluation of polymyxin B AUC/MIC ratio for dose optimization in patients with carbapenem-resistant Klebsiella pneumoniae infection
Source: Front Microbiol. 2023 Aug 22;14:1226981. doi: 10.3389/fmicb.2023.1226981 (PMC10477696; doi:10.3389/fmicb.2023.1226981)
Supplement: Supplementary file 1 [file Data_Sheet_1.docx]

**Table S1** Antimicrobial susceptibilities of carbapenem-resistant *Klebsiella pneumoniae* isolates (MICs, mg/L, n = 77).

| Antibiotics | Breakpoints | R (%) | I (%) | S (%) | MIC50 | MIC90 | MIC Range | Methods |
| --- | --- | --- | --- | --- | --- | --- | --- | --- |
| PMB | S≤2 R≥8 | 1.3 | 0 | 98.7 | 1 | 1 | 0.25-16 | BMD |
| COL | S≤2 R≥8 | 0 | 0 | 100 | 0.5 | 0.5 | 0.5-16 | Viket 2 |
| CAZ/AVI | S≤8 R≥16 | 2.4 | 0 | 97.6 | 2 | 2 | 0.064-256 | KB |
| CPZ/SBT | S≤16 R≥32 | 98.6 | 0 | 1.4 | 64 | 64 | 4-64 | KB |
| PIP/TAZ | S≤16 R≥128 | 98.6 | 0 | 1.4 | 128 | 128 | 2-128 | Viket 2 |
| CAZ | S≤4 R≥16 | 98.6 | 0 | 1.4 | 64 | 64 | 0.125-64 | Viket 2 |
| FEP | S≤2 R≥16 | 97.3 | 1.4 | 1.4 | 32 | 32 | 0.125-32 | Viket 2 |
| ATM | S≤4 R≥16 | 98.6 | 0 | 1.4 | 64 | 64 | 1-64 | Viket 2 |
| IPM | S≤1 R≥4 | 98.7 | 0 | 1.3 | 16 | 16 | 0.25-16 | Viket 2 |
| MEM | S≤1 R≥4 | 98.7 | 0 | 1.3 | 16 | 16 | 0.25-16 | Viket 2 |
| AMK | S≤16 R≥64 | 72.6 | 0 | 27.4 | 64 | 64 | 1-64 | Viket 2 |
| CIP | S≤0.25 R≥1 | 95.8 | 0 | 4.2 | 4 | 4 | 0.25-4 | Viket 2 |
| LVX | S≤0.5 R≥2 | 91.7 | 4.2 | 4.2 | 8 | 8 | 0.125-8 | Viket 2 |
| TMP/SMX | S≤2 R≥4 | 59.4 | 0 | 40.6 | 32 | 32 | 1-32 | Viket 2 |
| MNO | S≤4 R≥16 | 65.3 | 13.9 | 20.8 | 16 | 16 | 0.5-16 | Viket 2 |
| TGC | S≤2 R≥8 | 21.9 | 23.3 | 54.8 | 2 | 8 | 0.5-8 | Viket 2 |

BMD, broth microdilution; Viket 2, VITEK® 2 COMPACT automated system; KB, disc diffusion method; PMB, polymyxin B; COL, colistin; CAZ/AVI, ceftazidime/avibactam; CPZ/SBT, cefperazone/sulbactam; PIP/TAZ, piperacillin/tazobactam; CAZ, ceftazidime; FEP, cefepime; ATM, aztreonam; IMP, imipenem; MEM, meropenem; AMK, amikacin; CIP, ciprofloxacin; LVX, levofloxacin; TMP/SMX, trimethoprim/sulfamethoxazole; MNO, minocycline; TGC, tigecycline.

**Table S2** Univariate analysis for sample types.

| Variable | Bronchoalveolar lavage fluid (n = 63) | Other samples  (n = 14) ^a^ | *P* |
| --- | --- | --- | --- |
| Age, years | 57.0 (52.0-66.5) | 62.5 (37.0-64.0) | 0.073 |
| Male | 46 (73.0%) | 11 (78.6%) | 1.0 ^b^ |
| Weight, kg | 70.0 (60.0-70.0) | 67.5 (60.0-80.0) | 0.920 |
| BMI, kg/m^2^ | 23.1 (21.4-24.2) | 24.5 (18.9-25.8) | 0.845 |
| SOFA score | 7.0 (6.0-10.0) | 3.0 (2.0-7.0) | 0.008 |
| APACHE II score | 20.0 (17.5-23.0) | 17.0 (13.0-21.0) | 0.014 |
| Septic shock | 24 (38.1%) | 6 (42.9%) | 0.741 ^c^ |
| Mechanical ventilation | 51 (81.0%) | 3 (21.4%) | < 0.001 ^b^ |
| Laboratory data | | | |
| GFR, mL/min⋅1.73m^2^ | 105 (73.2-119) | 105 (94.8-126) | 0.400 |
| Albumin, g/L | 29.2 (28.0-31.9) | 31.6 (27.5-33.8) | 0.895 |
| Platelets, 10^9^/L | 182 (134-274) | 283 (117-464) | 0.736 |
| CRP, μg/L | 82.3 (32.5-166) | 72.9 (58.1-101) | 0.132 |
| Procalcitonin, ng/mL | 0.99 (0.27-3.32) | 1.05 (0.40-1.50) | 0.473 |
| Polymyxin B treatment | | | |
| Dose/weight, mg/kg/d | 1.88 (1.54-2.40) | 2.27 (2.0-2.50) | 0.551 |
| AUC_ss,24h_ | 48.3 (34.2-68.9) | 73.8 (36.5-103.5) | 0.267 |
| AUC/MIC | 46.5 (32.0-65.0) | 68.3 (43.8-99.1) | 0.253 |
| AUC/MIC ≥ 50 | 33 (52.4%) | 9 (64.3%) | 0.418 ^c^ |
| Clinical outcomes | | | |
| Response | 38 (60.3%) | 11 (78.6%) | 0.236 ^b^ |
| 30-day mortality | 24 (38.1%) | 5 (35.7%) | 0.868 ^c^ |

GFR, glomerular filtration rate; CRP, C-reactive protein; AUCss,24h, the area under the curve across 24 hours at steady state; MIC, minimum inhibitory concentration.

a, other samples included blood, cerebrospinal fluid, hydrothorax and ascite, and skin tissue pus.

b, Categorical variables were analyzed by Fisher's exact test.

c, Categorical variables were analyzed by Chi-square test.

**Table S3** Probability (%) of target AUC, PTA, and CFR for different polymyxin B regimens according to the 10^th^, 50^th^, and 90^th^ percentiles of creatinine clearance (CrCL).

| CrCL (mL/min) | Dosing regimen  (q12h) | Probability (%) of target AUC | Probability (%) of AUC > 100 mg·h/L | PTA for different MICs (mg/L) | | | | | | | CFR (%) for PK/PD target |
| --- | --- | --- | --- | --- | --- | --- | --- | --- | --- | --- | --- |
|  |  |  |  | 0.25 | 0.5 | 1 | 2 | 4 | 8 | 16 |  |
| 50.5 | 50 mg | 55.5 | 19.6 | 100 | 98.3 | 75.1 | 19.6 | 1.1 | 0 | 0 | 76.3 |
|  | 75 mg | 44.2 | 50.7 | 100 | 99.9 | 94.9 | 50.7 | 7.9 | 0.1 | 0 | 93.9 |
|  | 100 mg | 22.3 | 76.5 | 100 | 99.9 | 98.8 | 76.5 | 18.7 | 0.6 | 0 | 98.1 |
| 104.6 | 50 mg | 45.9 | 8.7 | 99.7 | 93.6 | 54.6 | 8.7 | 0.1 | 0 | 0 | 58.5 |
|  | 75 mg | 56.9 | 28.7 | 100 | 99.6 | 85.6 | 28.7 | 2.2 | 0 | 0 | 85.4 |
|  | 100 mg | 39.8 | 53.0 | 100 | 99.9 | 94.8 | 55 | 6.9 | 0.1 | 0 | 94.0 |
| 140.7 | 50 mg | 39.9 | 5.9 | 99.5 | 91.3 | 45.8 | 5.9 | 0 | 0 | 0 | 50.9 |
|  | 75 mg | 55.6 | 21.9 | 100 | 99.3 | 79.5 | 21.9 | 1.3 | 0 | 0 | 80.1 |
|  | 100 mg | 46.8 | 44.7 | 100 | 99.9 | 91.5 | 44.7 | 4.4 | 0 | 0 | 90.9 |

Target AUC, 50-100 mg·h/L; PTA, probability of target attainment; CFR, cumulative fraction of response; PK/PD target, PK/PD ≥ 50.


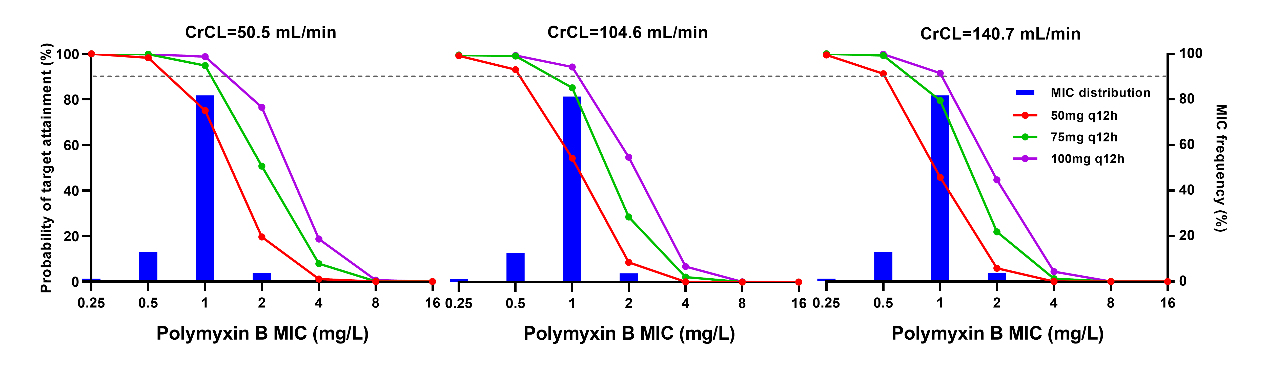


**Figure S1** Probability of target attainment (PTA) of various regimens with different creatinine clearance (CrCL). The target was the area under curve/minimum inhibitory concentration (AUC/MIC) ≥ 50. Histograms represent the distribution frequency by broth microdilution. Horizontal dotted lines represent 90% PTA.
